# Understanding Mind–Body Experience from the Perspective of Interoceptive Awareness: A 21-Day Embodied Practice Intervention

**DOI:** 10.3390/bs16030411

**Published:** 2026-03-11

**Authors:** Zixi Liu, Zhen Wu, Jingchao Zeng, Haosheng Ye

**Affiliations:** 1 School of Education (Teachers College), Guangzhou University, Guangzhou 510006, China; 2 Center for Mental Health Education and Counseling, Guangzhou HuaLi Science and Technology Vocational College, Guangzhou 511325, China; zyrf-wz@foxmail.com; 3 Department of Psychosocial and Psychoanalytic Studies, University of Essex, Colchester CO4 3SQ, UK

**Keywords:** mind–body relationship, flow experience, interoception, embodied cognition, breathing practice, cross-cultural perspective

## Abstract

This qualitative study examined how a 21-day integrated program fosters interoceptive awareness and mind–body integration among urban adults in mainland China (*n* = 11). The intervention combined daily nasal breathing regulation, spontaneous mandala making, and descriptive journaling, complemented by weekly group sharing. Using a cultural–psychological lens, we investigated how an inward–turning tradition in Chinese culture shapes embodied experience and meaning–making. Applying Interpretative Phenomenological Analysis to diaries, drawings, and focus-group data, we identified three interrelated processes: (1) the refinement of bodily attention; (2) a shift from deliberate control to natural immersion; and (3) the symbolization of feeling through artistic expression and social resonance. Findings indicate that systematic engagement in the “breath–mandala” intervention heightened sensitivity to chest-centered embodied sensations and promoted the integration of bodily experience into personal narratives; a non-goal-directed, relaxed practice style facilitated the transition from control to absorption, activating self-regulatory mechanisms; and non-evaluative awareness deepened flow while supporting cognitive reorganization and reflective capacity. The study delineates a core pathway by which breath-triggered interoceptive work operates within mind–body interventions, offering a theoretical basis and practical direction for tailored regulation programs across diverse populations.

## 1. Introduction

Contemporary mind–body research endeavors to explore how individuals maintain sensitivity to and regulatory capacity over internal bodily signals amidst fluctuating environments and complex self-narratives. This pursuit of “turning inward” not only draws deeply from the wisdom of traditional Chinese practices such as Daoist breath regulation and Chan (Zen) introspection (Pati & Zubko, 2019; Shi et al., 2020), but also resonates closely with advances in contemporary cognitive science. This line of inquiry leads us to the theoretical perspective of embodied cognition. This theory posits that cognition is not an internal representation of the world, but is enacted through the ongoing, recurrent sensorimotor coupling between an organism as an autonomous agent and its environment (Thompson & Stapleton, 2009; Varela et al., 1991). This implies that cognition, experience, and meaning emerge from the dynamic interaction between the body and the world (Thompson, 2005). Embodied cognition reframes the understanding of experiences such as emotions: they are no longer internal states awaiting interpretation, but modes of being that are instantly presented through the interaction of mind–body and environment (Clark, 1997). Within this framework, interoception—defined as the perception of internal bodily signals such as heartbeat, respiration, and visceral sensations—constitutes the bodily foundation for the regulation of emotion, attention, and action, and serves as a critical starting point for individuals to generate meaning through interaction with the environment (Ehmann et al., 2025; Eslinger et al., 2021; Hanley et al., 2017). As a core process through which individuals perceive and interpret internal bodily signals, interoception acts as a bridge connecting the body and the mind, and forms a key link in psychosomatic integration (Cameron, 2001; Quigley et al., 2021). For instance, the Polyvagal Theory proposed by Porges posits that the ventral vagal complex is closely associated with cardiopulmonary function, social engagement, and the regulation of a sense of safety, thereby laying a neuroanatomical foundation for the interaction between internal bodily sensations and psychological states (Porges, 1995). This indicates that interoception is not a single sensory pathway, but rather a dynamic process of perceiving, interpreting, and assigning meaning to internal signals (Khalsa et al., 2018; Schillings et al., 2022). It is involved not only in real-time safety-threat assessment, but also in supporting a broader range of experiences related to connectedness, creativity, and meaning-making (Porges, 2022).

Focusing on the cultivation of interoception, breathing emerges as an ideal practical entry point due to its unique physiological and psychological properties. From an embodied perspective, the conscious regulation of breathing constitutes an intervention that actively reshapes the perception-action cycle, thereby influencing the generation of experience. At the neural level, breathing forms a dynamic brain-body closed loop: bottom-up rhythmic signals generated in the brainstem ascend to higher-order centers such as the anterior insula and anterior cingulate cortex, while top-down inputs from these same brain regions modulate respiratory activity (Nakamura et al., 2024). Behavioral research indicates that slow breathing can suppress sympathetic overactivation and promote parasympathetic dominance, and rhythmic breathing is also associated with improvements in cognitive function (Chin & Kales, 2019; Zaccaro et al., 2018). Of particular importance is the tight link between respiratory phase mechanisms and interoceptive processing: inhalation tends to bias attention towards exteroceptive sampling, whereas exhalation facilitates interoceptive processing (e.g., by enhancing anterior insula activity). This provides a mechanistic basis for modulating interoception through breath regulation (Molle & Coste, 2022). Building upon these mechanisms, in practice, prolonged exhalation can be viewed as a form of interoceptive training. Under parasympathetic influence, it helps individuals cultivate neutral, non-judgmental bodily observation and recalibrate evaluative appraisal mechanisms (Liang et al., 2025; Weng et al., 2021). Existing research has demonstrated that lower interoceptive accuracy is associated with higher levels of anxiety, suggesting that biased interpretation of normal bodily signals may exacerbate emotional distress (Chemis et al., 2025). Recent studies have further integrated breathing exercises with descriptive journaling (see Supplementary Materials), guiding individuals to objectively record bodily sensations and emotional labels. This combined practice aims to strengthen non-evaluative attention to pre-reflective experience, thereby establishing a precise foundation of bodily self-awareness for more complex cognitive-emotional interactions (Liang et al., 2025).

This refinement of perception and enhancement of regulatory capacity fostered by breathing practice aligns closely with the experience of flow. Flow is described as an optimal experience characterized by the complete merging of action and awareness and deep attentional absorption, where the sense of enjoyment and autonomy originates intrinsically (Csikszentmihalyi, 1990; Jackson et al., 2023). From an embodied cognition perspective, flow represents the emergence of a highly coordinated perception-action coupling pattern, characterized by heightened action fluency and a temporary attenuation of self-referential evaluation (Leroy & Cheron, 2020; Shepherd, 2022; Swann et al., 2016, 2017, 2019). Therefore, cultivating interoceptive precision and self-regulatory capacity through breath training essentially lays the phenomenological and physiological groundwork for this more autonomous and immersive experiential mode.

However, a key theoretical and practical question arises: How can these finely tuned interoceptive sensations and potential flow experiences, often residing at a pre-reflective level and acquired through bodily training, be more clearly grasped by the individual and integrated into their ongoing world of meaning and life narrative? This requires a medium that can externalize and objectify embodied experiences, thereby allowing the individual to engage in a new dialogue with their own experience for further meaning generation. In this context, mandala making as an expressive arts practice shows unique value. In Jungian psychology, the mandala is regarded as an image that emerges spontaneously under conditions of affective tension, aiming to restore inner order and balance (Jung, 1963/1989, 1988). Spontaneous mandala making provides a symbolic medium through which pre-linguistic bodily feelings can be rendered visible, brought into awareness, and integrated (Cox, 2021; Fincher, 2009). Empirical research has largely developed along two paths: templated coloring has been shown to reduce anxiety, whereas spontaneous creation aligns more closely with the emphasis on self-expression and mind–body integration and is associated with nonverbal emotion regulation (Curry & Kasser, 2005; Wang, 2024; Zhu et al., 2025; see Supplementary Materials for definitions). Across populations, mandala-based interventions have been associated with improvements in emotion regulation and self-awareness, as well as reductions in anxiety and depressive symptoms (Dadashi et al., 2025; Kim et al., 2018; Liu et al., 2020; Yakar et al., 2021).

Although existing studies have confirmed the clinical utility of mandala creation, a descriptive gap persists in the current literature when viewed from the perspectives of embodied cognition and phenomenology. Most relevant studies focus on verifying intervention effects or elucidating symbolic integration functions, but provide limited in-depth phenomenological investigation into the subjective experiential process itself. The present study prioritizes the richness and authenticity of lived subjective experience over interpretive or symbolic analysis. Specifically, prior research has yet to adequately clarify how individuals cultivate interoceptive awareness through practices such as breath-focused attention (e.g., sensing bodily sensations and directing attention inward); what distinct subjective experiences arise during the transition toward flow states; and in what ways these pre-reflective embodied experiences subsequently shape cognition.

For the present study, this gap implies that practices which stabilize interoceptive processing (e.g., exhalation-focused breathing) and methods for externalizing pre-reflective experience (e.g., descriptive journaling and spontaneous mandala creation) can serve as valuable process-oriented materials. These approaches help elucidate how interoceptive and exteroceptive channels interact to reduce self-referential processing while preserving accurate behavioral guidance, ultimately facilitating entry into flow states (Goral et al., 2024; Seng & Group, 2019). Conceptually, we conceptualize flow as an embodied, immersive state characterized by complete engagement and absorption in the activity, rather than merely a cognitive or emotional label (Csikszentmihalyi, 1990; Jackson et al., 2023; Leroy & Cheron, 2020).

In response to this need for theoretical and practical integration, this study proposes and follows an integrated pathway: using embodied cognition as a meta-framework, we regard interoception (cultivated through breathing exercises and descriptive journaling) as the bodily foundation for experience generation, consider flow (characterized by effortless immersion) as a potentially emergent optimized action mode, and view spontaneous mandala making as the key practice for realizing the externalization and objectification of experience, thereby initiating a new cycle of meaning generation. We hypothesize that the systematic combination of breathing, descriptive journaling, and spontaneous mandala drawing can facilitate a continuous generative process: from the refinement of bodily perception, to a tendency towards immersive action patterns, and further to the creative externalization of internal experience and its reflective reconstruction.

### Aims and Objectives

This study aims to explore how participants experience body awareness through somatic practices within an intervention framework combining breathing exercises and mandala making. Specifically, the study seeks to investigate how individuals, through the enhancement of interoceptive awareness, achieve autonomous regulation of the body and mind. The intervention framework is designed such that breathing exercises primarily help participants increase their awareness of internal bodily experiences, while the descriptive journaling assists them in accurately recording these experiences. Mandala making, as a daily focused practice, provides participants with a way to express and experience these sensations. Although the primary goal of the intervention is to enhance interoceptive awareness and promote somatic regulation, how participants spontaneously transition from body awareness to flow states (optimal experiences) through these practices will gradually be revealed during the research process. Through this framework, the study will focus on how the activation of interoception facilitates participants’ reflection and psychological transformation.

The core research questions of this study are as follows (see Table 1):

RQ1: How do participants’ experiences change over the course of 21 days when their attention shifts from external events to internal bodily states?

RQ2: In what ways do participants conceptualize and articulate these experiences following the activation of interoceptive awareness?

RQ3: How does the accumulation of these experiences facilitate their reflective processes and cognitive transformation?

## 2. Method

### 2.1. IPA Theoretical Foundations

Interpretative Phenomenological Analysis (IPA) is grounded in phenomenology and hermeneutics, offering a qualitative methodology that emphasizes understanding the subjective experiences of individuals. Phenomenology, as the philosophical foundation of IPA, focuses on examining lived experiences by bracketing assumptions and focusing on how the world appears to the individual. It enables a detailed exploration of personal experiences in their raw form, ensuring that the researcher engages with the world as it is experienced by the participant. Hermeneutics, on the other hand, introduces an interpretative layer, recognizing that understanding human experience is inherently interpretative. In IPA, researchers are involved in a “double hermeneutic”, where participants interpret their own experiences, and the researcher interprets these interpretations. This dual process of interpretation makes IPA particularly suited to exploring complex phenomena such as interoceptive awareness and somatic experiences, where understanding is built on how individuals make sense of their bodily sensations (Biggerstaff & Thompson, 2008; Smith et al., 2009).

This combination of phenomenology and hermeneutics makes IPA an ideal methodology for exploring the interaction between mind and body. The process of interoceptive awareness, where individuals attune to their internal bodily sensations, is inherently subjective and dependent on how the individual makes sense of these sensations. IPA allows for a deep exploration of how participants perceive, articulate, and reflect on their somatic experiences, making it a robust tool for investigating embodied cognition and mind–body integration (Biggerstaff & Thompson, 2008; Robinson & Williams, 2024).

In this study, IPA provides a method to explore how breathing exercises and mandala making activate interoceptive awareness, and how this activation influences participants’ psychological transformation. By focusing on individual narratives and the meaning-making process, IPA allows the researcher to examine the complex, dynamic relationship between bodily perception and psychological experience, providing an appropriate framework for analyzing body–mind interactions (Robinson & Williams, 2024).

### 2.2. Study Design and Intervention Process

This study adopted an Interpretative Phenomenological Analysis Focus Group (IPA-FG) design to explore participants’ subjective experiences in depth. Central to this approach is positioning participants as experts, guiding them to articulate their physical and mental experiences authentically and reflect on them, thereby fostering meaning construction (Cutrer-Párraga et al., 2024; Love et al., 2020).

The focus group interviews were designed to cultivate a collective learning environment for the systematic exploration of lived experiences and cognitive expansion. To minimize biases inherent in group dynamics, such as conformity, a two-round independent speaking protocol was implemented: an initial round for personal statements based on individual experiences, followed by a supplementary round after all members had been heard, with no cross-discussion permitted. This structure aimed to safeguard the depth of individual expression and psychological safety. All sessions were facilitated by a qualified professional trained in psychological counseling and group facilitation.

Aligned with IPA’s emphasis on researcher reflexivity (Smith, 2019), the research team assumed distinct roles: the first author, who organized the discussions, navigated dual insider-outsider identities; the second author provided observational support. Although not present during the focus groups, the third author and the fourth author contributed critically to the research design by interrogating the theoretical framework and intervention plan. Furthermore, the third author conducted a comprehensive review of all qualitative data—including interview transcripts, drawings, and diaries—following data collection.

**Figure 1:**
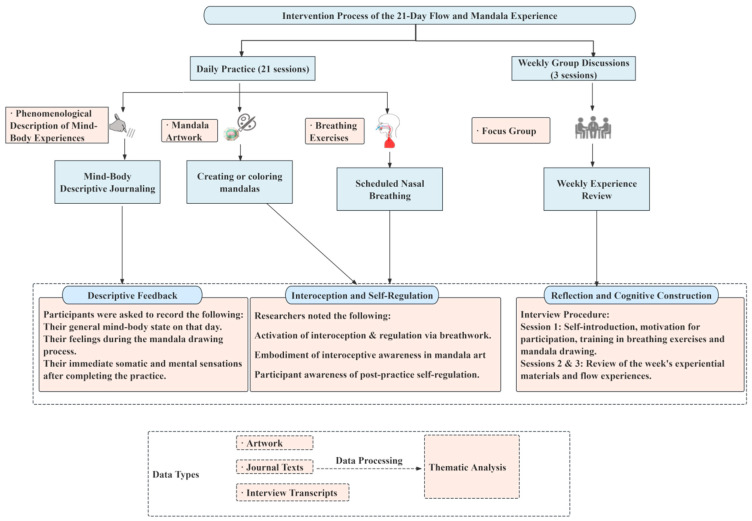
21-Day Mind–Body Integration Intervention Flow.

This intervention employs a 21-day integrated program of nasal breathing exercises and mandala making designed to enhance participants’ interoception, self-regulation, and emotional awareness (see Figure 1). Throughout the process, participants progressively strengthen their awareness and regulatory capacity over physiological, emotional, and psychological states.

During daily practice, participants were required to engage in a 10 min fixed-duration nasal breathing exercise (with emphasis on complete exhalation; see Supplementary Materials for the guiding instructions) and mandala creation (with no fixed time limit for the creation process; see Supplementary Materials for the guiding instructions). In addition, participants were advised to voluntarily practice nasal breathing in daily life according to their own needs, so as to enhance bodily awareness and facilitate the expression of mind–body experiences. Its value lies in providing participants with an open, non-judgmental space for expression, allowing mind–body experiences to unfold naturally—which aligns with the essence of flow as “wu-wei” (non-action) and the core connotation of spontaneous mind–body integration. Following each session, participants document changes in their physical and mental states before, during, and after practice, with particular attention to bodily sensations and their impact on cognitive and emotional experiences. This documentation helps participants identify internal shifts, thereby further promoting self-regulation and a deeper understanding of their mind–body condition.

Weekly group discussions provide a platform for participants to share their practical experiences. These discussions focus on bodily sensations and emotional changes arising during practice, without addressing specific life events, and emphasize how mind–body changes influence personal cognition and emotional states. Such sharing not only enables participants to gain deeper insight into their own mind–body state but also enhances their understanding of their motivation for engaging in the activities and the self-regulation process, while creating space for reflection on prior cognitive patterns.

The core of the intervention lies in integrating self-regulation practices with mandala making to help participants enhance awareness of their mind–body state and foster spontaneous mind–body integration. Through breathing exercises and mandala creation, participants experience the flow of internal energy and the release of emotions. These changes gradually deepen their understanding of their internal state and cultivate a more positive outlook on life.

### 2.3. Participants

Eleven participants were recruited through the WeChat social platform. During the recruitment process, potential participants were informed about the study’s objectives and were invited to contact the first author if they were willing to engage in a 21-day activity and could consistently complete the associated tasks. The inclusion criteria for participants were as follows: (1) aged 18 or older, (2) native Chinese speakers, and (3) able to express themselves through writing. All participants were residents of mainland China. The recruitment process was conducted in multiple phases, with three groups of participants being recruited: the first group (*n* = 4) in January 2024, the second group (*n* = 4) in June 2025, and the third group (*n* = 5) completing the recruitment in the same year. In total, 11 participants completed all activities and interviews (2 participants did not complete the full activities). The participants’ ages ranged from 23 to 54, with 4 male participants and 7 female participants (see Table 2).

Before the interviews, participants completed a questionnaire about their experience with flow states and a survey regarding their recent physical and mental health. These were used to gather basic demographic information, assess whether participants had previously been aware of or experienced flow states, and exclude those with any significant recent physical or mental health issues. The specific exclusion criteria included participants who reported severe mental health problems (e.g., depression, anxiety) or significant physical health issues in the survey. The first author provided all participants with a detailed explanation of the basic concepts of flow (see Section 1) and the relevant practice methods, including breathing exercises and the descriptive journaling technique (emphasizing faithful, as-is description). Participants were informed that there were no right or wrong ways to approach the mandala making and descriptive journaling exercises, and that there were no fixed standards of evaluation; they were encouraged to express their experiences based on their personal state. Additionally, the first author provided professional support for any technical issues related to the nasal breathing exercises during the study to ensure that participants could successfully engage in the practices.

Following the completion of the 21-day intervention and the final focus group interviews, the research team subsequently implemented aftercare follow-up procedures to support the participants’ physical and mental well-being. At the end of the last group session, the participants’ efforts and contributions were acknowledged and appreciated by the research team. The team explicitly informed the participants that if they experienced any discomfort or had any questions for clarification after the intensive interoceptive practice, they could contact the first author. This support channel remained open for four weeks after the formal conclusion of the study. No participant reported needing additional professional psychological support beyond the aforementioned communication channel during the study implementation and the subsequent follow-up window.

### 2.4. Ethics

This project was conducted in accordance with the Declaration of Helsinki and obtained ethical approval from the Research Ethics Committee of Guangzhou University (No. XYZ123). All participants provided online informed consent prior to the study, with explicit acknowledgment of their right to withdraw at any time. Following data collection, participants verified the accuracy of interview transcripts and reconfirmed consent for academic publication of their anonymized data. All procedures adhered strictly to the approved ethical protocol and relevant professional guidelines.

### 2.5. Data Analysis

This study conducted complete audio and video recordings of the focus group discussions to observe the influence of contextual emotions and actions. All interview recordings and diary entries were transcribed verbatim, and the participants’ artwork was also archived on a case-by-case basis. In terms of data analysis, this study adopted the Interpretative Phenomenological Analysis (IPA) steps proposed by Cutrer-Párraga et al. (2024). This method is particularly suited for focus group data and fully considers both within-case and across-case analysis, which aligns well with the data collection methods of this study. The specific analysis process is as follows:

(a) Repeatedly reading the original transcripts to become familiar with the data content;
(b) Systematically annotating and coding each focus group transcript;
(c) Maintaining a reflexive stance to clarify how the researchers’ own perspectives affect the interpretation;
(d) Developing and consolidating the emerging categories to construct a preliminary analytical framework;
(e) Refining the categories into higher-level themes to capture the core meanings of the data;
(f) Repeating the above steps for each case to gain a deeper understanding of individual experiences and identify final themes;
(g) Conducting across-case comparisons to inductively derive common patterns and themes;
(h) Maintaining reflexivity throughout the process to minimize subjective bias as much as possible.

After the initial reading of the transcripts, the research team sifted through the data and excluded segments that were not relevant to the research goals. The first and second authors independently coded all the text, and then they compared and discussed the codes with the third author. This division of labor was designed to integrate different perspectives (e.g., internal and external roles) for a more diverse understanding of the material. For example, some researchers focused more on the differences between participants’ interoceptive awareness and their previous cognitions, while others concentrated on capturing the dynamic development of body and mind states through mandala making and texts. During reflexive discussions, the team deeply explored the potential cognitive biases brought by the researchers’ identities, clarified the impact of their stances on interpretation, and acknowledged the value of multiple perspectives in deepening the understanding of the data.

Building on this, the research team integrated the preliminary codes into more generalized categories, gradually approaching the “core essence” of participants’ original embodied experiences (Alase, 2017). Each member carefully read the text and marked key content and typical examples to ensure rigor and transparency in the analysis process. Through systematic coding, categorization, and theme refinement, the final thematic structure was formed, reflecting the process of change in participants’ interoceptive awareness. This also identified common patterns across the groups. These patterns revealed the evolution of participants’ experiences and the construction of meaning in aspects such as body perception, mental health, self-cognition, and social relationships.

### 2.6. Reflexivity

This study adheres to the principles of Interpretative Phenomenological Analysis (IPA), which emphasizes sustained researcher reflexivity. To minimize the potential impact of researcher bias, the team implemented systematic measures across all stages of data collection and analysis. The research team comprised four members: three held doctoral degrees in psychology (including a professor of educational psychology), and one was a psychology lecturer. Both intervention facilitators possessed local mental health practitioner qualifications and substantial group counseling experience. The lead facilitator had an extensive background in training and teaching the relevant techniques, ensuring intervention fidelity. All participants were engaging with the program for the first time.

Given that during the intervention phase, the first author’s theoretical understanding of interoceptive awareness and mind–body integration might have unconsciously shaped the framing of guidance provided to participants, thereby leading participants’ expressions of their experiences to align with the exploratory hypotheses of the study (facilitation bias) and further introducing bias into the interview and interaction processes, the present study adopted the following measures to mitigate such bias as much as possible:

Each focus group was assigned a facilitator and an assistant. To clarify roles and mitigate researcher influence, the facilitator explicitly outlined the process at the outset, emphasizing that their role was strictly limited to providing technical guidance on breathing exercises and sharing related experiences—not to evaluate or advise on participants’ personal experiences. Furthermore, group discussions were structured in a non-interactive format without cross-talk among participants. This approach aimed to reduce the facilitator’s perceived authority, foreground the interview-like (rather than instructional) nature of the discussion, and encourage candid sharing of personal experiences. For process monitoring, the assistant solicited and provided feedback on the facilitator’s guidance before and after each session. The facilitator subsequently debriefed with the assistant and maintained a reflective journal to record personal emotions, cognitions, and contextual observations. These journals served as materials for in-depth examination of potential biases during thematic analysis.

This study critically interrogates both the facilitative and constraining dynamics of the focus group format in generating phenomenological data. A key limitation arises from the potential for conformity effects: despite the implementation of independent speaking protocols, participants’ articulations of embodied experience may inadvertently converge toward group consensus, thereby undermining phenomenological authenticity. To mitigate this risk, a three-week period of individual practice and reflective journaling was introduced prior to the group sessions, thereby minimizing prior interpersonal influence. Conversely, the format’s strength lies in its ability to foster a distinctive emotional context and a secure containing space: shared narratives among participants help elicit and refine subtle or ambiguous embodied sensations that are often difficult to access in individual interviews.

Bias may also arise during the data processing phase of this study: due to the first author’s prior familiarity with participants’ practice processes and personal experiential narratives, the objectivity of coding and thematic interpretation might be compromised, with the risk of overinterpreting data consistent with the research framework and underemphasizing contradictory or divergent experiential descriptions (interpretation bias). Therefore, during the data analysis phase, a triangulation strategy was employed to enhance objectivity and rigor. Colleagues not involved in the intervention provided critical feedback: experts independently reviewed and questioned the research design and outcomes, while the third author reviewed all interview materials and participated in the full IPA process. Through mutual verification across facilitator, assistant, and external expert perspectives, this study sought to identify and reduce subjective bias at the methodological level, thereby strengthening the credibility and interpretive rigor of the findings. A triangulation check revealed no significant subjective bias in this study; only a small number of coding discrepancies existed, which were revised following collective discussion by the research team.

It should also be noted that although this study has minimized the potential bias caused by the first author’s dual role through a series of targeted strategies, it must be objectively acknowledged that the subjective influence of researchers participating in both intervention implementation and data analysis cannot be completely eliminated, which is also an inherent limitation of qualitative intervention research.

## 3. Results

Through an Interpretative Phenomenological Analysis (IPA) of participant interviews, this study identified three superordinate themes and four subordinate themes (see Table 3), which systematically illustrate how the activation of interoceptive awareness reshaped their mind–body experiences. These findings directly address the core research questions: through the 21-day practice of breathing and mandala making, as participants shifted their attention from external events to their internal bodily states (RQ1), they underwent a transformation from somatic blockages to energy flow (Theme 1) and progressed from conscious control to a state of “effortless doing” and flow immersion (Theme 2). Building upon this foundation, they conceptualized and articulated these experiences through artistic externalization (Theme 3a) and social resonance (Theme 3b) in response to RQ2. Detailed descriptions are provided in the Supplementary Materials. Ultimately, this process fostered reflection and integration that extended from personal cognition to interpersonal relationships (RQ3). The following sections will elaborate on this internal trajectory of transformation in detail, supported by extensive participant excerpts and visual examples from their artwork.

### 3.1. Theme 1: The Embodied Starting Point: From Perceiving Blockages to Generating Experiential Space

The majority of participants in this study initially reported perceiving physical blockages such as fatigue, tension, and exhaustion in their mind–body experiences. As the intervention progressed, they transitioned from passively enduring these discomforts to actively engaging with breathing exercises to anchor and heighten their bodily awareness. Through descriptive journaling, participants shifted their focus from proliferating thoughts and external distractions to their internal bodily sensations. As a result, they frequently experienced a loosening of previously fixed somatic blockages, observing a fluid transformation in their internal states. This progression naturally led to the emergence of a tangible and inhabitable “flow experience space”.

Participants commonly experienced a positive shift from bodily sensations of “blockage,” “tightness,” and “exhaustion” to respiratory smoothness and internal flow. This experiential transition effectively compensated for the somatic and psychological depletion caused by long-term, high-intensity mental work, marking a clear shift from a state of fatigued tension to one of relaxed fluidity.

LC (Group 3), a typical knowledge worker, found that breathing exercises offered more than mere relaxation; they constructed a fluid mind–body pathway leading from a state of physiological exhaustion towards inner tranquility and clarity. As his practice deepened, his perception of internal energy shifted from being “congested in the head” to “flowing freely throughout the entire body,” forming a cool and light internal environment. This transformation is consistently reflected in his diary entries: “*My body heats up and sweats every day during practice, my mind feels calm*” (Day 6); “*An itch between my eyebrows, feeling energy spinning there… my brain is continuously being cleansed (by a water-like energy sensation)*” (Day 9); “*The airflow is like a quiet stream flowing inside my body, cool and fresh, washing over my blood vessels and brain*” (Day 20); “*While breathing, it feels like sitting on a cloud, my body is light, breathing is smooth, warm currents spread from the top of my head to my face, arms, and torso*” (Day 21).

XR (Group 1), a secondary school teacher struggling with stress-induced insomnia, described in the final interview a state of dissipated fatigue achieved through breathing exercises.

XR: *Once (while doing breathing exercises)… I felt an emptiness between my collarbone and ribs, my body felt very light, my thoughts were completely clear, and both my heart and body felt empty. I believe that in a flow state, an individual may merge with the entire environment, becoming one unified whole.*

Interviewer: *Is this your personal experience?*

XR: *Yes.* (Week 3)

XR mentioned that after experiencing the merging of the individual with the overall environment and the emptiness of mind and body, she created her final mandala work (day 21). She then stated that she simply wanted to depict the light in space (see Table 4).

JH (Group 2) found it difficult to describe the experience of fatigue release after completing breathing exercises. In Week 1, he stated:

*This feeling is hard to describe because right now I’m actually very calm. It seems like nothing can stir my emotions… My mind is very quiet… I just tried to focus my attention on the area of my chest… How to describe it? It’s like there’s a spring there, constantly bubbling up. The edges (of the chest) feel inflated, but the center is empty.*

By Week 3, his experience became more embodied:

*Suddenly it felt as if… a tap had been turned on. I located the position, roughly right here (pointing to the center of the chest) and up about this much. Then, from this central point upwards, it kept flowing, just like mountain spring water, continuously flowing downwards. Very icy, very cool… It just flowed down and then stopped. After that, I couldn’t really hear what was being said anymore, so I just kept focusing on that sensation.*

Beyond these cases, YY, TZ, and FQ also described flow channels located in the chest, head, or spreading upwards from the lower limbs. Notably, participants differed in their characterization of the flow’s quality: JH, LC, and FQ more often described the substance within the channels as cool or warm water flows, whereas TZ, XR, and YY depicted a flow more akin to air, which was notably experienced as pushing through physical and mental blockages. Regarding spatial perception, LC and XR reported experiences of spatial awareness extending beyond the body’s boundaries, integrating with or feeling infinitely close to the environment. In contrast, in their earlier diary entries, TZ, JH, and LC more frequently used “tubes” and “balloons” as metaphors to portray the form, texture, and dynamics of internal energy.

XR (Group 1) described it as “*like a deflated balloon being slowly inflated bit by bit… from the outside in… the sense of control gradually strengthens*”. Similarly, SS (Group 2) experienced “*a balloon with a hole, constantly expanding… the sensation of energy spreading throughout the body*.” She also observed changes in the “tube” within her body: “*Sometimes the tube is very thick… constantly expanding… very large. Now it’s in that thin, tight state… it still exists in my body*”. JH (Group 2) used a “*swimming ring*” to metaphorize his mind-body state, describing it as “*tight around the edges and empty in the middle*.” TZ (Group 2) felt “*I was trembling, continuously trembling, starting roughly from the diaphragm, then outward, layer by layer. First to the lower abdomen, then the thighs, then upward to the back, shoulders… finally reaching the hands*.” FQ (Group 3) experienced “*the airflow rising from the soles of my feet to my abdomen*.” JJ (Group 3), while breathing with eyes closed, felt “*energy right at the top of my head, scalp, and around my eyes… feeling the air flowing outward. It’s a bit itchy and tingling*.” As his practice deepened, his bodily awareness became increasingly refined, enabling him to acutely detect experiential differences in temperature, flow, tension, and emptiness across various body regions.

In later practice stages, WZ discovered that focused breathing itself could regulate his smoking cravings, with the urge and related physical discomfort significantly alleviated (Week 3):

*Generally, in daily life, it’s hard for me to go half an hour without smoking. Right now, I completely don’t have that (feeling)… but once I stop the practice, the craving immediately returns… still scratching (the respiratory tract)… if I don’t consciously do the breathing practice, it still scratches… that thing (the claw-like object) is still there, it’s just that doing the breathing practice helps it pass through.*

WZ’s practical journey demonstrates a transformation path based on bodily perception. This case reveals the potential value of interoceptive mapping in addiction intervention, illustrating how body-based work can provide new regulatory pathways for habitual behaviors.

### 3.2. Theme 2 Moving Beyond Purpose: From Control to “Effortless Doing” in Immersion

In the initial phase, as participants were still familiarizing themselves with the intervention, a goal-oriented approach was prevalent. They attempted to achieve a flow state by concentrating on completing the breathing exercises and mandala making. This very purposeful effort, however, often inhibited full immersion in the present moment. With deepening practice and accumulated experience, they gradually relinquished expectations and control, naturally entering a state of immersion. This shift in mental models outlines a clear trajectory from purposeful striving to aimless relaxation and effortless engagement. The activation of interoception and the occurrence of flow states subsequently promoted a broader transformation in their cognitive patterns, fostering a move away from preset notions and judgment toward greater openness and a grounding in direct experience. The following two sub-themes will explore how this transition from control to effortlessness unfolded through their personal accounts.

#### 3.2.1. Theme 2a: The Transition from Striving Control to Natural Immersion

This sub-theme reveals the participants’ shift in identity from “actor” to “experiencer.” In the early stages, most participants exhibited a goal-oriented state focused on “effort” and “completion.” However, this striving mindset often triggers tension in interoception, thereby hindering the continuity of immersive experiences.

XY (Group 1) initially approached the practice with a “task-participation” mindset, showing clear signs of urgency—such as frequently checking the time, worrying about personal performance in social interactions, and even experiencing “chest tightness” due to nervousness during her first focus group session (Week 1). This initial discomfort gradually became a focus of her self-breakthrough as the practice progressed. She began to experience full immersion in breathing and drawing, making it possible to “draw to her heart’s content, losing track of time.” She wrote in her journal:

*I felt more engaged in the breathing training and more adaptable… Today, I didn’t stop drawing when the time was up; I kept going until I finished what I wanted to draw, without even realizing how much time had passed.*

(Day 3)

In the second-week interview, XY further reflected on the relationship between focus and experience:

*Today, I truly felt a sense of calm and relaxation. In previous breathing sessions, sometimes I could feel it, but if my mind was preoccupied and I wasn’t focused on the practice, that sense of flow would disappear.*

When asked how she achieved focus, she recognized its non-controllable nature:

*It doesn’t feel like something I control—it’s a state. Sometimes, I just can’t focus… Currently, in daily life, I experience more focus and full engagement. But I feel that achieving flow in everyday life still falls short compared to during breathing exercises; the sense of flow seems stronger in the breathing practice.*

By the third week, XY’s transformation had extended to the interpersonal level:

*It just occurred to me—in terms of interpersonal interactions, after these 21 days, I feel I care much less than before… During our first discussion, I found self-disclosure exhausting, but now I hardly feel that way. … I can also perceive the connections between others.*

She described this connection as an embodied “flow” experience:

*The transmission of emotions, the surge of feelings. …It’s hard to describe—like a warm flow (emanating from my chest). It’s truly a novel experience, so I’m still figuring out how to articulate it.*

SS (Group 2) provided a detailed account of the “control-relaxation-immersion” process, emphasizing that the awakening of interoception requires moving beyond a task-oriented approach and that the subsequent state of immersion arises spontaneously, rather than being achieved through prior striving:

*In the first few minutes of exhaling, I could clearly feel myself exerting effort to push the breath out. But in the following minutes, it gradually became easier… (When drawing mandalas) At first, my mind was still occupied with things like color matching. Later, I naturally stopped thinking about anything and entered that state completely. I became fully immersed and finished the drawing, feeling as though time had flown by.*

(Week 2)

*One insight I’ve had is that I believe we shouldn’t approach this with a goal in mind. I need to feel first, to empty my mind, to enter that state of emptiness, and then slowly begin to observe. I need to see what changes this brings me. I can’t do this with a purpose—that’s my small realization.*

(Week 2)

**Figure 2:**
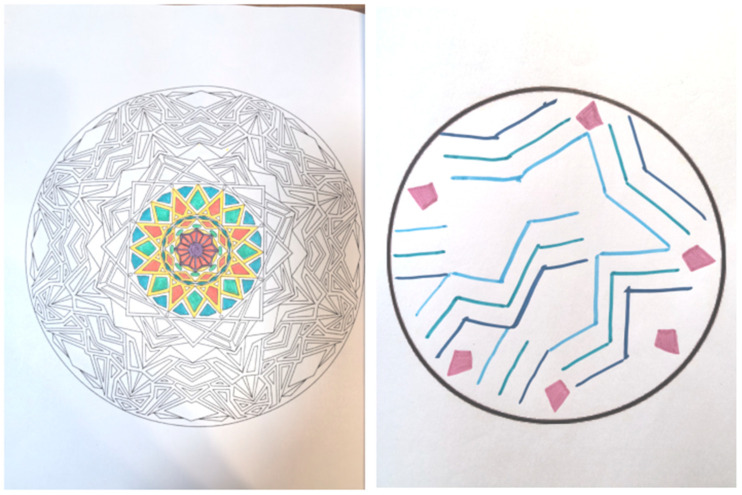
A comparison of LC’s mandala making: Day 11 ((**left**), incomplete) and Day 12 (**right**). (This image externalizes an embodied sensation: the mandala’s flowing blue lines wash over purple energy points, mirroring the participant’s repeated experience—recorded in their diary (Days 9, 11, & 12)—of a watery flow coursing through the space between the eyebrows and the brain).

LC (Group 3) transferred his “achievement-oriented” mindset from work to his drawing practice. His early journal entries repeatedly noted anxiety caused by “(mandala patterns) being too complex to finish in half an hour” (Day 3), leaving him “anxious, tense, and unable to focus on the present” (Day 6). After the second focus group discussion, he began to “let go of the pressure,” viewing the practice as a process rather than a task. This shift toward non-purposefulness brought a sense of mental lightness, making immersive experiences possible. On Day 11, he did not complete that day’s drawing, and from Day 12 onward, he spontaneously chose simpler patterns for his creations (see Figure 2). In his Day 12 journal, he wrote: “During breathing practice, I had more saliva in my mouth. The itching sensation between my eyebrows spread to my nose and chin. I felt focused and calm, and my body warmed up. (Although) external stimuli heightened my anxiety, I could regulate it with breathing. In the evening, I felt irritable because my child wouldn’t sleep. After drawing, I felt better.” This record indicates that his focus had shifted from outcome completion to process regulation, with his internal state transforming accordingly.

The participants gradually relaxed their tension and need for control over outcomes, yet continued to engage consistently in practice and daily life from a more relaxed state. This process of self-regulation—from “control” to “relaxation”—effectively resolved the tension between purpose and experience, ultimately guiding them toward interoceptive experiences they had never encountered before or had never been clearly aware of.

#### 3.2.2. Theme 2b: The Paradox of Expectation: The Beginning and Disruption of Deep Experience

Several participants clearly articulated how “expectation” paradoxically served as both the summoner and terminator of deep experiences. This tension between conscious intention and lived experience reveals an essential quality of profound states: they cannot be directly commanded by the conscious mind; in fact, conscious control often disrupts the natural emergence of such states.

FQ (Group 3) provided a vivid example of this paradox:

*Then (as I practiced the breathing exercises), although lying in bed, I could no longer feel the bed—only myself. Then I sensed an energy flow, rising continuously from the soles of my feet upward, until it nearly reached my abdomen. I very much hoped it would keep rising all the way to my head, but then it suddenly cut off, and I could no longer find that sensation.*

(Week 3)

This stands in stark contrast to her earlier experience:

“*This week I’ve been extremely busy, and the more I tried to enter that state, the less I could access it. That time last week, I was completely relaxed, and it [the sensation of the energy flow rising gradually] suddenly emerged on its own*”.

(Week 3)

TZ (Group 2) also recognized the interference of goal-directed intention: “*I feel there’s some obstruction now… It feels like certain attachments are beginning to arise… It’s because I ‘want to achieve it,’ I have this thought.*” When the interviewer asked, “*So when you have this goal, it actually doesn’t work as well*?” he confirmed, “*…Yes*.” *To the follow-up question*, “*So you can’t achieve the same level of wholehearted immersion as before*?” he again replied, “*…Yes*.”

Other participants described how their preconceived notions about the flow experience itself shaped their expectations and, consequently, limited the diversity of their experiences. WZ (Group 2) expressed in early interviews a prior assumption that flow was a state of “high emotional arousal” and “quick reflexes.” Consequently, he excluded the “low-energy,” “calm” states experienced during breathing practice from his definition of flow. As his practice deepened, he experienced and came to acknowledge another form of flow, characterized by “immersion-relaxation-focus,” thereby reconstructing his understanding of the optimal state.

*I’ve done some self-reflection… After a week of practice, I feel it’s actually not about high arousal… I feel that state is very focused, but within it, I’m actually settled down, not like the heightened state I’m in during ordinary tasks—it’s different… (After experiencing both states) I feel that after completing tasks in this calm state, there’s actually little [fatigue], whereas if it were a consistently high-arousal state, I’d feel quite tired afterward.*

(Week 2)

XR (Group 1)’s journal entry (Day 2) also reflected the dissolution of such preconceptions:

*I originally thought the ‘optimal state’ required ‘mental exertion,’ but through direct experience, I discovered it’s about a relaxed mind. During mandala making, my breathing gradually slowed, and my mind grew increasingly calm and relaxed.*

Their narratives subtly reflect a shift in mindset—a gradual reduction in obstacles caused by preconceived notions and expectations. Notably, the repeated mentions of “feeling” in this section are not accidental: this phenomenon precisely indicates that the antecedents of cognitive restructuring stem from prior embodied experiences. This suggests that “feeling” typically refers to raw, embodied experiences (e.g., calmness, concentration, a sense of flow), while cognitive restructuring is the subsequent reflective meaning-making process through which participants interpret, integrate, and act upon these embodied experiences. This sequential relationship aligns closely with the core premise of the present study: interoceptive awareness (encompassing both bodily sensations and emotional feelings) serves as the foundation for mind–body integration and cognitive transformation, rather than deliberate striving toward cognitive goals. Ultimately, the attainment of deep experience begins precisely with letting go of the deliberate expectation of “wanting to achieve it”—as many participants have recounted, when they cease clinging to the goal of “reaching a certain state,” raw embodied experiences emerge spontaneously and lay a solid foundation for subsequent cognitive restructuring.

### 3.3. Theme 3. The Generation and Resonance of Meaning: From Artistic Externalization to Interpersonal Connection

Through the combined practice of breathing exercises and mandala making, participants’ engagement evolved from initial emotional regulation to a meaning-generating process encompassing somatic, psychological, and social dimensions. mandala making served as a pivotal symbolic medium—its colors, lines, and creative gestures formed an embodied language, enabling participants to give form to vague internal sensations and emotional fluctuations, transforming them into visual expressions that could be articulated, examined, and shared, thereby bringing forth, integrating, and releasing content previously beyond words.

When these works, laden with personal experience, were articulated and explored within the group context, individuals’ inner narratives transcended their initially nebulous personal perceptions, extending into a shared space open to collective recognition and discussion. Within this space, through mutual listening and reflection, individuals experienced a sense of “flow” connection and social belonging. Thus, the construction of meaning progressed from internal self-repair toward interpersonal integration and resonance, demonstrating that interoceptive awareness not only deepens self-understanding but also creates a developmental field. This field allows individuals to explore the congruence between their interoceptive experience and social identity, facilitating an integrative and expressive process of growth.

#### 3.3.1. Theme 3a: Giving Form to the Unspeakable: Mandala as an Expression of Inner States

Mandala making served as a vital medium for participants to give tangible form to their internal experiences: they translated sensations of energy, emotions, and thoughts into colors, lines, and imagery, allowing originally vague interoceptive sensations to acquire perceptible and shareable forms.

The case of YY (Group 1) demonstrates the profound connection between the creative process and bodily experience. With prior experience in introspection and meditation, YY chose daily mandala creation as a means of dialoguing with her mind–body state. Beginning from her seventh artwork, a symbolic dot gradually emerged at the center of her compositions (see Table 5). Concurrently, having studied chakra knowledge, she began reporting sensations of blockage in her heart chakra area, accompanied by emotional fluctuations linked to traumatic memories. She described this experience as “the pain of a wound being torn open.”

When reflecting on her mandala creations during this period, YY described her creative process:

*(The painting process) has nothing to do with my conscious thinking… they’re all completely improvisational… it’s a state that naturally emerges in the present moment… Yes, I can now answer the question you asked me earlier—(about the dot that consistently appeared in the center of my mandala over several days) Is there a correlation with my physical sensation (chest tightness)? Yes, there is indeed a connection. I feel I’ve come to understand it now.*

(Week 2)

*(When sharing trauma-themed artwork) I felt embarrassment and shame… but I unloaded those fears… I feel I was very courageous.*

(Week 2)

When YY used mandala making to express her embodied pain, she realized this artistic expression resonated profoundly with her physical state. The process of giving concrete form to inner wounds initially brought feelings of shame and unease, but after sharing, she experienced a sense of relief as if a heavy burden had been lifted from her shoulders (Week 2). YY regards mandala making as a sacred space that transcends ordinary daily experience. Here, spontaneous, improvisational creation becomes a ritual for externalizing emotions and trauma: transitioning from “bloody, mangled” wounds to nourishment through “unconditional love.” For her, mandala creation is not merely an artistic form but a ritual space for healing and revelation—where suppressed emotions find expression, blocked energy finds release, and the soul finds refuge.

JJ (Group 3), when reflecting on a special experience in her painting practice during the final stage, expressed:

*You know what? I often have this feeling—I truly feel that the brush tip, that very fine stroke (though the brush we actually use is much thicker). When you paint over the paper, it normally might not reach into such fine crevices. But I can, and I just paint like this, feeling that the brush tip carries ‘energy,’ you understand? Yes. And originally I didn’t know what the flow state was, but while painting, the brush tip feels just like myself, you know? Like, for every shape’s boundary—sometimes after I outline the form, painting inside feels particularly joyful. You can stay within the boundaries yet paint very freely. That swishing sound feels particularly comfortable, particularly exhilarating. At those moments, the brush tip, that ‘energy’—makes me feel so… I think this must be similar to (the flow experience).*

(Week 3)

#### 3.3.2. Theme 3b: The Social Construction of Meaning: Cognitive Restructuring Through Shared Experience

The focus group interactions powerfully demonstrated how meaning is co-constructed within a shared experiential space. When the same intervention was refracted through diverse personal perspectives, it yielded a rich spectrum of distinct yet equally authentic and profound experiences. As participants externalized the various internal experiences they had realized during practice and brought them into the group setting, their personal narratives began to intersect and resonate within this collective container, catalyzing a process of deep cognitive restructuring.

XY’s (Group 1) experience illustrated a shift from personal cognition to social connection. She found that in interpersonal interactions she “didn’t care as much as before,” and could feel genuine joy witnessing the growth of her peers. Within the group, she experienced a transpersonal emotional flow:

*Because XR can relax through her drawing, looking at her artwork and descriptions makes me feel I can empathize with her experience… It’s like feeling happy for someone who has recovered from a cold… Another point is… I feel there’s a flow within our group… Because only when the emotions expressed by one person resonate with another can this feeling of flowing connection arise… I feel (the location of this sensation) is the heart. Because when I wasn’t thinking but just listening to those words, there was a flowing sensation in the area of the heart…*

TZ (Group 2) described how he empathized with SS’s resilient optimism in facing hardship, stating:

*While SS was speaking, I felt as if she helped remove that wall (in the chest)… Then after a while I felt quite happy. Just felt happy listening to her share… It was a feeling of positivity… It seems what she shared wasn’t entirely positive; there were some negative feelings too. But what I felt was happiness.*

LC (Group 3) began reflecting on and adjusting his cognitive patterns during the practice, realizing a “shift in mindset: no need to push myself so hard in everything.” The practice became a field for observing and adjusting his relationship with himself, moving from “compulsion” to the more flexible “doing my best.” Recalling past deep flow experiences, LC understood that the essence of flow is deep integration with the environment (“very close”) and inner tranquility, not merely efficient thinking:

*During junior high… that state of studying… somewhat like what ZY just described, as if everything around had stopped, but my feeling was slightly different, yet similar. It wasn’t that it stopped; the surroundings felt very close, but time, if you compare time to flowing water, it was still flowing. But it was very serene, calm, you could feel it flowing. Yet you were fully focused and wholeheartedly in your own… that kind of learning, just happy anyway. Don’t know (how to describe it)… In that state, thinking of things was very easy, learning was very easy, also very efficient.*

(Week 3)

This retrospective examination of purely cognitive flow made LC realize that the essence of flow lies in complete engagement and connection. He further reflected that this state of oneness with an activity might not be confined to moments of solitary problem-solving, but could also exist within profound connections between people. It was based on this insight that he extended the flow experience to the interpersonal realm during the final focus group.

In the final focus group, while describing his relationship with his wife, he extended the flow experience to the interpersonal realm: “Could similar states also exist in relationships between people? For example, my relationship with my wife is free of distractions, very secure, and of course, free of cluttering thoughts…… Just now, when I described my relationship with my wife, I realized my heart was becoming more and more excited…” (Week 3). His description reflects a flow experience within interpersonal relationships: in safe, trusting relationships, one can also experience focus, peace, and a mutually nourishing state.

LC’s accounts of flow states in scholarly pursuits and personal relationships acted as a mirror, illuminating ZY’s own predicament. This brought her to the crucial understanding that her central challenge now was how to “find her way back” from a condition of being torn by external judgments, and re-access that deeply immersive flow which springs from a solid, inner stability: “Just now, during LC’s sharing, I also recalled (my junior high days)… I was just very focused on listening to the teacher’s expression itself… I noticed others didn’t seem to be like me, and then I felt that back then it was actually a focused, immersed flow state, completely indifferent to scores and such. Recalling those scenes, I feel my heart back then could be so steady… Later, gradually it became less and less steady, don’t know why I abandoned all that… later the influence from the environment or others’ swaying became significant. Of course, I can’t blame others entirely; I myself also swayed back and forth over trivial matters. Not knowing what to do, just making myself annoyed.”

These interactions collectively wove a collaborative web of meaning-making: individual experiences were validated and amplified within the group, enabling participants to refine their understanding of self, others, and the world through mutual reflection. Ultimately, the self-practice of nasal breathing exercises, descriptive journaling writing, and mandala making not only facilitated personal transformation but also cultivated a relational epistemology—a realization that our fundamental connection to the world and others is a given, varying only in our degree of awareness. By nurturing this inner sensibility, we come to perceive this woven web of relations from a new perspective; this awareness is the prerequisite for our ability to face the world and make choices, and it is also the foundation guiding the individual toward deeper integration with the whole.

## 4. Discussion and Clinical Implications

In Theme 1, nasal breathing exercises (with emphasis on complete exhalation) guided participants to shift their attention inward. Most participants initially noticed physical fatigue and tension, then gradually experienced a sense of flow within internal “channels,” accompanied by the alleviation of physical discomfort. A few participants further reported experiences of bodily integration with space—although the external environment might be stressful, their internal space felt tranquil and expansive. Compared to the localized improvement in fatigue brought by the internal flow sensation, this holistically unfolding spatial experience led to a comprehensive mind–body transformation, thereby addressing RQ1’s focus on the shift in attention from external to internal bodily experiences. Furthermore, the awareness of channels, flow, and space also prompted changes in individual cognition. As interoceptive sensations entered conscious awareness, participants’ experiences of certain behaviors (e.g., smoking) differed from before, triggering reflections on past states and leading to adjustments in behavior and cognition. This aligns with RQ3’s exploration of how accumulated experiences facilitate reflection and cognitive transformation.

In Theme 2, the study revealed a trajectory of participants transitioning from “striving for control” to “natural immersion.” Initially, many participants approached the practice with a task-oriented mindset, attempting to “achieve” a flow state through willpower, which inadvertently hindered the natural emergence of immersive experiences (2a). As the practice deepened, they gradually recognized the “paradox of expectation” (2b): the more they pursued profound experiences, the more they disrupted their natural flow. This finding resonates with core characteristics of flow theory, such as the “balance between challenge and skill” and “spontaneous engagement” (Csikszentmihalyi, 1990), and illustrates how the activation of interoceptive awareness drives shifts in cognitive patterns (RQ3). Participants realized that only by relinquishing control and engaging relaxedly could genuine flow experiences emerge. In doing so, they naturally abandoned previously controlling coping strategies, transitioning from “goal-driven” to “process-oriented” engagement. At this stage, transcendent experiences such as “blockages being cleared by the wind” spontaneously occurred, and long-standing physical and mental struggles dissolved.

In Theme 3, mandala making served not only as a medium for externalizing inner states (3a) but also as a vehicle for shared meaning-making and interpersonal connection within the group (3b). Through colors, lines, and composition, participants gave tangible form to otherwise ineffable bodily sensations and emotional states, achieving cognitive restructuring and emotional resonance during group sharing. This process demonstrates how raw, experiential knowledge is transformed into understandable and integrable cognitive value. From an objective standpoint, when individuals are situated in a group setting, their feelings naturally interact with others, and regardless of whether these interactions are positive or negative, they inevitably influence the participants. This process of “social construction of meaning” not only strengthened individual reflective awareness but also fostered interpersonal connection and empathy, highlighting the potential of mind–body integration interventions to promote harmony at both individual and societal levels.

### 4.1. Clinical Implication

A salient finding is that participants’ reports consistently highlighted the chest region, describing it as a core focus of enhanced interoceptive awareness. Observations revealed that participants often first and most vividly perceived dynamic bodily sensations—such as blockages and flows—in this area. This suggests that the chest may function as a critical mind–body interface during the process of psychosomatic integration (Yang, 2023, p. 18).

Within a theoretical context, the Polyvagal Theory provides a potential physiological reference framework for the pervasiveness of this experience. This theory posits that the ventral vagal complex, which regulates social engagement behaviors and feelings of safety, is closely linked to the heart and lungs both anatomically and functionally. It is important to note that we do not equate the participants’ reported sensations of “openness,” “flow,” and “connection” in the chest directly with the activation of the ventral vagal complex. Instead, we argue that this neurophysiological framework can offer a perspective for understanding the physiological underpinnings of bodily experiences (Porges, 1995, 2022).

From both clinical and Chinese cultural perspectives, the significance of the chest (or the “heart region”) extends far beyond its role as a mere physiological site. In the context of Chinese culture, it is regarded as the abode of the heart-spirit and an embodied symbolic vessel that carries emotions, subjectivity, and spiritual states (Yang, 2023, p. 40). Participants’ descriptions of chest sensations are inherently metaphorical: “blockage” symbolizes unresolved emotional distress, while “flow” metaphorically represents the harmony and integration of mind–body states. Turning to narrative cases, YY associated chest tightness with emotional trauma, whereas XY, TZ, and LC extended their chest-centered experiences to interpersonal relationships. These narratives collectively demonstrate that bodily sensations in the chest are not isolated physiological signals, but rather symbolic experiences deeply intertwined with core elements of self-narrative (including emotion, trauma, and social connection), whose connotations are shaped by cultural cognition and personal meaning.

In summary, from the perspective of embodied cognition, breathing exercises and descriptive journaling refined participants’ interoceptive sensitivity toward the chest region—a process that embodies interoception as a “dynamic meaning-making process.” This transformation turned the chest from an often-neglected site of tension into a dynamic space for self-perception through embodied interaction. Meanwhile, mandala making and expression, as tools for externalizing embodied experiences, further facilitated self-regulation via the interaction between the body and the symbolic environment, aligning with the core proposition of embodied cognition that meaning is generated through the dynamic interaction between the body and the world (Varela et al., 1991). Crucially, participants were not passive experiencers; they actively engaged with somatic sensations in the chest as a tangible interoceptive anchor, using this bodily foundation to reorganize and integrate personal narratives concerning work, addiction, trauma, and relationships. This underscores that the enhancement of interoceptive awareness is not merely the strengthening of physiological perception, but a deeply personal and meaning-laden constructive process within the framework of embodied cognition—one in which the chest acts as a pivotal interface for mind–body interaction, serving as the core vehicle for individuals to perceive the world and construct the self through bodily experience.

The findings of this study offer the following implications for mental health promotion and group intervention practice:

Interoceptive Training as a Foundation for Mind–Body Integration: Breathing exercises and body description diaries help enhance individuals’ emotion regulation and self-awareness, making them particularly suitable for contexts such as anxiety relief, stress management, and post-traumatic growth. By systematically guiding participants to focus on internal bodily sensations, this intervention provides a feasible pathway for the embodied processing of emotions and cognition.

The “Non-Purposive” Design Fosters the Development of Self-Regulation Capacity: This intervention emphasizes experiential process over goal achievement, without prescribing a standardized definition of flow or evaluating outcomes. This design creates a psychological space for free exploration, enabling participants to develop personalized regulation strategies in a non-directive environment. Compared to interventions targeting specific conscious-level issues, this study focuses more on cultivating participants’ intrinsic self-regulatory capacity, an approach that holds significant value for promoting long-term psychological adaptation.

Social Resonance Mechanisms in Group Processes: The non-interactive sharing design used in the focus groups ensures psychological safety for individuals while facilitating a collective healing process of “resonance–restructuring.” By listening to others’ experiences, participants gain emotional resonance and cognitive inspiration. This social learning mechanism is applicable to group therapy and mental health education courses, providing an actionable framework for constructing supportive group environments.

### 4.2. Research Limitations and Future Directions

This study has several limitations. First, the small sample size and predominance of highly educated participants restrict the generalizability of the findings. Second, it is important to clarify that this is an exploratory qualitative study, whose core contribution lies in describing the experiential processes of mind–body integration rather than verifying the causal effects or efficacy of the intervention. In line with this research orientation, additional limitations include constraints associated with the focus group design and potential biases arising from researcher involvement. Third, although data were collected from three groups at different time points, further validation is required to determine whether the intervention-related experiential patterns are applicable across diverse cultural contexts or whether they may vary when delivered by different facilitators. It is recommended that future studies adopt mixed-methods approaches, combining physiological indicators with qualitative data, to more comprehensively capture the mechanisms of mind–body interventions. Additionally, long-term follow-up studies would help examine whether such experiences lead to sustained psychological transformation.

## 5. Conclusions

Based on the implementation and observation of a 21-day mind–body integration intervention, this study draws the following conclusions:

(1) Systematic engagement in breathing and mandala creation significantly enhanced participants’ interoceptive awareness. This enhancement was specifically manifested in participants’ increased sensitivity to embodied sensations in the chest region, along with a gradual proactive integration of these bodily experiences into personal narratives. This aligns with the core view in the Introduction that “interoceptive awareness serves as a key starting point for connecting mind and body and generating meaning,” thereby validating breathing as an ideal entry point for cultivating interoception. It demonstrates that breathing can reshape the perception-action cycle through dynamic mind–body interactions.
(2) The non-goal-oriented and relaxed practice approach played a pivotal role in linking bodily awareness with flow experience, effectively facilitating participants’ transition from conscious control to natural immersion, and thereby activating self-regulatory mechanisms of both mind and body. This finding directly responds to the hypothesis in the Introduction that “flow is an embodied optimal experience that requires precise interoceptive awareness as its foundation,” while corroborating the practical logic that “deliberate control hinders the natural emergence of experience.” It resonates with traditional Chinese inward-exploration wisdom, such as Daoist breath regulation and Chan (Zen) introspective contemplation.
(3) The non-judgmental awareness cultivated during the intervention not only deepened participants’ flow experiences but also further promoted positive transformations in their cognitive patterns and enhanced reflective capacity. This result addresses the core question raised in the Introduction—“how pre-reflective embodied experiences are transformed into meaning construction”—and confirms the unique value of mandala creation as a medium for externalizing experience, thereby realizing a complete generative process from refinement of bodily perception to cognitive restructuring.

From the perspective of embodied cognition, this study reveals the core pathway through which breath-triggered interoceptive work operates in mind–body integration interventions: breathing training activates interoceptive awareness as the foundation, non-goal-oriented practice bridges to flow experience, and mandala creation achieves experiential externalization and meaning construction. This pathway not only echoes the core proposition of embodied cognition in the Introduction—“cognition, experience, and meaning arise from the dynamic interaction between body and world”—but also provides a theoretical foundation and practical direction for developing tailored mind–body regulation programs to meet the needs of diverse populations.

## Figures and Tables

**Table 1:** Research Questions, Theoretical Constructs, and Corresponding Operational Indicators.

Research Questions,	Theoretical Constructs	Operational Indicators (Data Sources and Measurement Methods)
RQ1	Interoceptive Awareness (Awareness of Internal Bodily Sensations)	1. Breathing Practice Record: The descriptive journaling documenting bodily states before, during, and after each daily practice. 2. Bodily Depiction in Interviews: Descriptions of changes in bodily sensations discussed during focus group interviews. 3. Mandala making: Expression of mind–body states through the use of color, line, and composition.
	Flow Experience (The optimal experience of immersion, loss of self, and focused attention)	1. Experience Descriptions in Interviews: Participants’ reports of optical performance emerging during drawing or breathing practices. 2. Process Observation: Researchers’ observation of participants’ attentional shifts and spontaneity during group activities. 3. Diary Texts: Records of moments during practice when the sense of time vanished and immersion emerged.
RQ2	Conceptual Presentation of Experience (Transforming Inner Experiences into Expressible and Shareable Forms)	1. Mandala making: Analysis of how participants’ descriptions of their artwork reflect and concretize inner states. 2. Descriptive journaling Texts: Records and labeling of bodily sensations and the metaphors used to describe them. 3. Focus Group Statements: How participants verbally describe, interpret, and share their bodily and emotional experiences.
RQ3	Meaning Construction/Reflective Awareness (Positive Changes in Cognitive Patterns, Self-Understanding, or Values)	1. Reflective Narratives in Interviews: Sharing new perspectives on past events and transformations in life attitudes. 2. Evolution of Diary Texts: Comparing diary entries across different time points to analyze changes in cognition, emotion, and self-understanding. 3. Transcendent Experiences and Social Connectedness: Instances of reported transcendent experiences or changes in interpersonal understanding arising from empathy and mutual support within the group.

**Table 2:** Case-related Information.

	*n* = 11
**Age**	
23–24	4
25–35	3
35–45	3
45–54	1
**Gender**	
Male	4
Female	7
**Education**	
Bachelor’s degree	2
Master’s degree	9
**Occupation**	
Graduate student (Educational Studies)	4
Secondary & High school teacher	2
University Lecturer	2
Pediatrician	1
Psychotherapist	1
Digital Influencer	1

**Table 3:** Superordinate and Subordinate Themes.

Theme	Sub-Themes
Theme 1: The Embodied Starting Point: From Perceiving Blockages to Generating Experiential Space	
Theme 2: Moving Beyond Purpose: From Control to “Effortless Doing” in Immersion	2a. The Transition from Striving Control to Natural Immersion
	2b. The Paradox of Expectation: The Beginning and Disruption of Deep Experience
Theme 3: The Generation and Resonance of Meaning: From Artistic Externalization to Interpersonal Connection (see Supplementary Materials)	3a. Giving Form to the Formless: Mandala as an Expression of Inner States
	3b. The Social Construction of Meaning: Cognitive Restructuring through Shared Experience (see Supplementary Materials)

**Table 4:** XR: Final Mandala and Descriptive Journal Entries.

Mandala Making	Description of Psychological State	Description of Physical State	Additional Statements
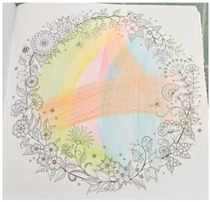	Fully immersed with no thoughts, just focused on drawing, feeling strength, freedom and joy.	I did many things and felt physically tired, but I entered a state of flow both in the morning and the afternoon.	simply wanted to draw the light in space.

**Table 5:** YY’s Mandala Artworks and Corresponding Mind–Body State Descriptions.

Mandala Making	Description of Psychological State	Description of Physical State	Additional Statements
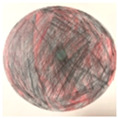	A matter that hurt my heart and I have not been able to let go of to this day, and I drew this emotion. Quite touching.	Feeling of blockage in the chest area	Before drawing, I was hesitant about this topic; while drawing, I hoped to let go little by little. Sharing it now, I hope the wound will be gently healed.
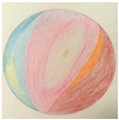	I thought of my mother, my dear mom, I was deeply held in her arms, feeling safe, trusting, and full of unconditional love.	Full, rich	Before drawing, I did a deep meditation, and I felt completely cleared. The inner child was seen and healed. After drawing, it became clearer.
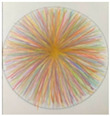	Drawing radiating lines from the outside to the inside, feeling the strength from the outside to the inside.	(In the process of) Experiencing at 0:00 on the 25th	The more I draw, the more I feel the abundance of the external environment stimulating my inner vitality.

## Data Availability

The datasets generated and analyzed during the current study are available from the corresponding author upon reasonable request.

## Supplementary Materials

The following supporting information can be downloaded at: https://www.mdpi.com/article/10.3390/bs16030411/s1, Definitions of Key Terms.
