# Supplementary material for: Understanding Mind–Body Experience from the Perspective of Interoceptive Awareness: A 21-Day Embodied Practice Intervention
Source: Behav Sci (Basel). 2026 Mar 11;16(3):411. doi: 10.3390/bs16030411 (PMC13023601; doi:10.3390/bs16030411)
Supplement: Supplementary file 1 [file behavsci-16-00411-s001.zip › behavsci-4032095-supplementary.pdf]

Supplementary Material: Definitions of Key Terms

To ensure terminological consistency and clarity throughout the manuscript, the following key terms are defined as used in this study:

Nasal breathing exercises: Put your tongue against the roof of your mouth, take a comfortable posture (either sitting or lying down), exhale through the nose until it is exhausted, stop naturally for a moment (don't hold your breath), inhale when you can't stand it anymore, and repeat the above process until the end of the exercise.

Mandala making: The spontaneous, non-prescriptive creative process of producing circular symbolic designs, allowing free expression of embodied sensations (e.g., warmth, tightness, flow) through colors, lines, and forms, without predefined patterns, artistic evaluation, or judgment of "right/wrong." This term encompasses the open-ended, expressive nature of the practice in our intervention and aligns with contemporary mandala-based art therapy literature (e.g., Wang, 2024; Zhu et al., 2025).Prior to the formal intervention, the research team provided participants with a clear introduction to mandala making:

"Mandala making is a way to 'speak' with your body design patterns in advance. Just let your brush follow the bodily sensations (e.g., warmth, tightness, or flow) you feel during breathing exercises, and express them freely through colors and lines."

"There are no right or wrong, good or bad evaluations for your works. The only requirement is to be honest with your current feelings what you draw is a true reflection of your mind-body state at that moment."

"The goal of this practice is to help you better perceive and integrate your inner experiences, not to complete a 'perfect' work. You can choose any colors or forms that resonate with you, without being restricted by rules."

Descriptive journaling: The non-judgmental, faithful documentation of bodily sensations, emotional states, and inner experiences before, during, and after each practice session, emphasizing objective recording over interpretation or analysis (e.g., Liang et al., 2025).

Social resonance: The immediate, empathetic attunement and shared emotional vibration among group members during sharing sessions, serving as the initial relational layer where individual embodied experiences begin to connect interpersonally through mutual recognition and emotional mirroring (e.g., Eslinger et al., 2021; Love et al., 2020; Porges, 2022).

Interpersonal connection: The emergent outcome of enhanced social bonding, mutual understanding, sense of belonging, and relational security, arising from accumulated resonance and shared meaning-making in the group context .(e.g., Kim et al., 2018; Liu et al., 2020; Porges, 2022)

Social construction: The collaborative, intersubjective process through which meanings of embodied experiences are negotiated, built, and transformed within the group, turning private bodily sensations into shared narratives that support cognitive, emotional, and relational integration (e.g., Cutrer-Párraga et al., 2024; Love et al., 2020).

Emotion regulation: The processes by which individuals modulate their emotional experiences, including their onset, intensity, duration, and expression. In this study, it is primarily embodied and interoceptive: arising from heightened awareness of internal bodily signals (especially chest-centered sensations of tightness, blockage, flow, or openness), supported by nasal breathing (promoting parasympathetic dominance and non-judgmental observation), descriptive journaling (objective recording without interpretation), spontaneous mandala making (nonverbal externalization of pre-reflective feelings), and group sharing (via social resonance and shared narratives for relational integration). This facilitates the shift from deliberate control to natural immersion (flow), activates intrinsic self-regulatory mechanisms, alleviates discomfort, and enables cognitive and emotional integration into personal narratives. This aligns with literature on interoceptive and mind-body pathways to emotion regulation (e.g., Liang et al., 2025; Porges, 1995, 2022; Wang, 2024; Weng et al., 2021; Zhu et al., 2025).

Cognitive restructuring: refers to the reflective, meaning-making process through which individuals interpret, integrate, and act upon embodied experiences and emotional feelings to achieve psychological transformation and adaptive cognitive patterns (Eslinger et al., 2021). In the context of this study, it is not a disembodied, top-down challenge of irrational thoughts, but a bottom-up, sequential process scaffolded by prior interoceptive and emotional awareness. Emotional feelings serve as raw, embodied antecedents (e.g., joy, relief, tension in the chest region), while cognitive restructuring follows as the interpretive and integrative step—participants reframe experiences (e.g., shifting from achievement-oriented to process-focused mindsets, extending flow to interpersonal resonance), thereby supporting mind-body integration and cognitive change. This aligns with contemporary embodied cognition research, where cognitive transformation is deeply rooted in and triggered by embodied sensations rather

than isolated rational deliberation (Eslinger et al., 2021; Hanley et al., 2017).
